# Complementary Roles of Short and Long Pentraxins in the Complement-Mediated Immune Response to *Aspergillus fumigatus* Infections

**DOI:** 10.3389/fimmu.2021.785883

**Published:** 2021-11-18

**Authors:** Raffaella Parente, Valentina Possetti, Marco Erreni, Francesca D’Autilia, Barbara Bottazzi, Cecilia Garlanda, Alberto Mantovani, Antonio Inforzato, Andrea Doni

**Affiliations:** ^1^ Istituto di Ricovero e Cura a Carattere Scientifico (IRCCS) Humanitas Research Hospital, Milan, Italy; ^2^ Department of Biomedical Sciences, Humanitas University, Milan, Italy; ^3^ The William Harvey Research Institute, Queen Mary University of London, London, United Kingdom

**Keywords:** *Aspergillus fumigatus*, aspergillosis, innate immunity, pentraxins, complement

## Abstract

The ubiquitous mold *Aspergillus fumigatus* is the major etiologic agent of invasive aspergillosis, a life-threatening infection amongst immune compromised individuals. An increasing body of evidence indicates that effective disposal of *A. fumigatus* requires the coordinate action of both cellular and humoral components of the innate immune system. Early recognition of the fungal pathogen, in particular, is mediated by a set of diverse soluble pattern recognition molecules (PRMs) that act as “ancestral antibodies” inasmuch as they are endowed with opsonic, pro-phagocytic and killing properties. Pivotal is, in this respect, the contribution of the complement system, which functionally cooperates with cell-borne pattern recognition receptors (PRRs) and other soluble PRMs, including pentraxins. Indeed, complement and pentraxins form an integrated system with crosstalk, synergism, and regulation, which stands as a paradigm of the interplay between PRMs in the mounting and orchestration of antifungal immunity. Following upon our past experience with the long pentraxin PTX3, a well-established immune effector in the host response to *A. fumigatus*, we recently reported that this fungal pathogen is targeted *in vitro* and *in vivo* by the short pentraxin Serum Amyloid P component (SAP) too. Similar to PTX3, SAP promotes phagocytosis and disposal of the fungal pathogen *via* complement-dependent pathways. However, the two proteins exploit different mechanisms of complement activation and receptor-mediated phagocytosis, which further extends complexity and integration of the complement-pentraxin crosstalk in the immune response to *A. fumigatus*. Here we revisit this crosstalk in light of the emerging roles of SAP as a novel PRM with antifungal activity.

## Introduction

Aspergillosis is a collective name for *Aspergillus* species-related infections that clinically manifest as either non-invasive (i.e., allergic bronchopulmonary aspergillosis, ABPA, chronic pulmonary aspergillosis, CPA, and aspergilloma), or invasive diseases (1). Invasive aspergillosis (IA) is the most severe form, with 10 million individuals at risk, more than 200,000 deaths/year worldwide, and a mortality rate of up to 90% in the worst scenarios (https://www.aspergillus.org.uk/). Several factors contribute to the risk and severity of IA, including microbial virulence, limited therapeutic pipeline and diagnostic inaccuracy, however it is the host’s immune status that primarily determines onset and progression of IA, with immune-compromised individuals being the most vulnerable (2).

IA is mainly caused by *Aspergillus fumigatus* (AF), an obligate aerobic filamentous fungus that spreads in the environment in the form of quiescent airborne spores (dormant or resting conidia) (3). Up to a few hundred spores are inhaled by humans daily, and, in immune competent individuals, most of them are mechanically eliminated by the ciliated and mucus-secreting cells of the epithelial barrier of the upper airways (4). Those who skip mucociliary clearance are promptly recognized, phagocytosed and killed by alveolar epithelial cells (mostly, type II pneumocytes) and cellular effectors of the innate immune system, including resident alveolar macrophages (AMs) and dendritic cells (DCs) as well as recruited polymorphonuclear neutrophils (5). These cells are all endowed with an armamentarium of pattern recognition receptors (PRRs) that recognize a spectrum of pathogen associated molecular patterns (PAMPs) on fungal spores, and activate mechanisms of defence (6). Neutrophils are particularly important in this respect, indeed iatrogenic, acquired and inherited defects in number, function or homing of these cells are major risk factors for IA (7).

Recognition and disposal of fungal particles are also mediated by soluble effectors of innate immunity, including complement, an ancestral system of soluble and cell-borne pattern recognition molecules (PRMs) (8, 9). Other PRMs are known to functionally cooperate with complement in the handling of AF infections, including ficolins, collectins and pentraxins. In particular, the long pentraxin PTX3 is an established complement-dependent PRM with host protective functions against AF [reviewed in (10)]. We have recently reported that the classical short pentraxin serum amyloid P component (SAP) promotes recognition, phagocytosis and killing of AF. However, this occurs through different complement-dependent mechanisms (11), which highlights complexity and integration of the innate immune reaction to fungal pathogens. Here, we discuss the pentraxin-complement interplay in IA, with a major focus on the most recent evidence from *in vitro* studies, animal modeling, and human genetics.

## Pentraxins and Their Interaction With the Complement System

Pentraxins are a superfamily of phylogenetically conserved proteins with regulatory functions in inflammation (12). C-reactive protein (CRP) and SAP, prototypes of the short pentraxins arm of the family, opsonize microbial pathogens and apoptotic cells, thus acting as soluble PRMs towards pathogen and danger associated molecular patterns, and support their complement-mediated clearance (13). CRP comprises 5 non-glycosylated subunits (14), whereas SAP is a plasma glycoprotein with 5 or 10 protomers (15). Both proteins share a peculiar quaternary structure with homo-oligomers folding into pentameric rings stabilized by non-covalent interactions (16).

In addition to CRP and SAP, PTX3 is a typical long pentraxin, with a C-terminal domain homologous to the short pentraxins, and an N-terminal region with no similarity to other proteins. The human PTX3 is a 340 kDa glycoprotein (17) made of 8 identical protomer subunits folding into a disulphide bond-stabilized octamer (18, 19). The amino acid sequence of PTX3 is highly conserved across species, suggesting an evolutionary pressure to preserve its structure/function relationships (20).

Despite these structural similarities, CRP, SAP and PTX3 are different in terms of cellular producers and molecular inducers. CRP and SAP are mainly synthesized in the liver, in response to IL-6 (21). CRP, whose serum concentration increases as much as 1000 times (from baseline levels of 0.8-1 mg/L) during acute responses, is the prototypic acute phase protein in humans and clinically used as a sensitive, though non-specific, systemic marker of infection and inflammation. The serum concentration of the murine protein however mildly increases (up to ~17 mg/L from baseline levels of 5-9 mg/L) upon LPS injection, which points to different mechanisms of gene regulation in the two species (22). CRP recognizes microbes and apoptotic cells by binding to phosphocoline (PC), and promotes phagocytosis of the opsonized materials through activation of the classical pathway (CP) of complement (21). In addition, CRP restrains complement hyperactivation by binding to factor H (fH), major inhibitor of the alternative pathway (AP) (23). SAP is an acute phase protein in mouse [with serum concentrations of ~500 μg/L and ~20 mg/L in homeostatic and inflammatory conditions, respectively (11)], whereas it is constitutively present in the human plasma (30-50 mg/L), where it contributes to host defence *via* direct or indirect (complement-dependent) opsonic mechanisms (24). Similar to CRP, SAP binds C1q (recognition unit of the CP) and promotes complement activation (25). While unable to bind fH, SAP interacts with C4b-binding protein (C4BP) (25, 26), major inhibitor of the CP pathway, indicating that, like CRP, SAP has complement regulating properties (27).

**Figure 1:**
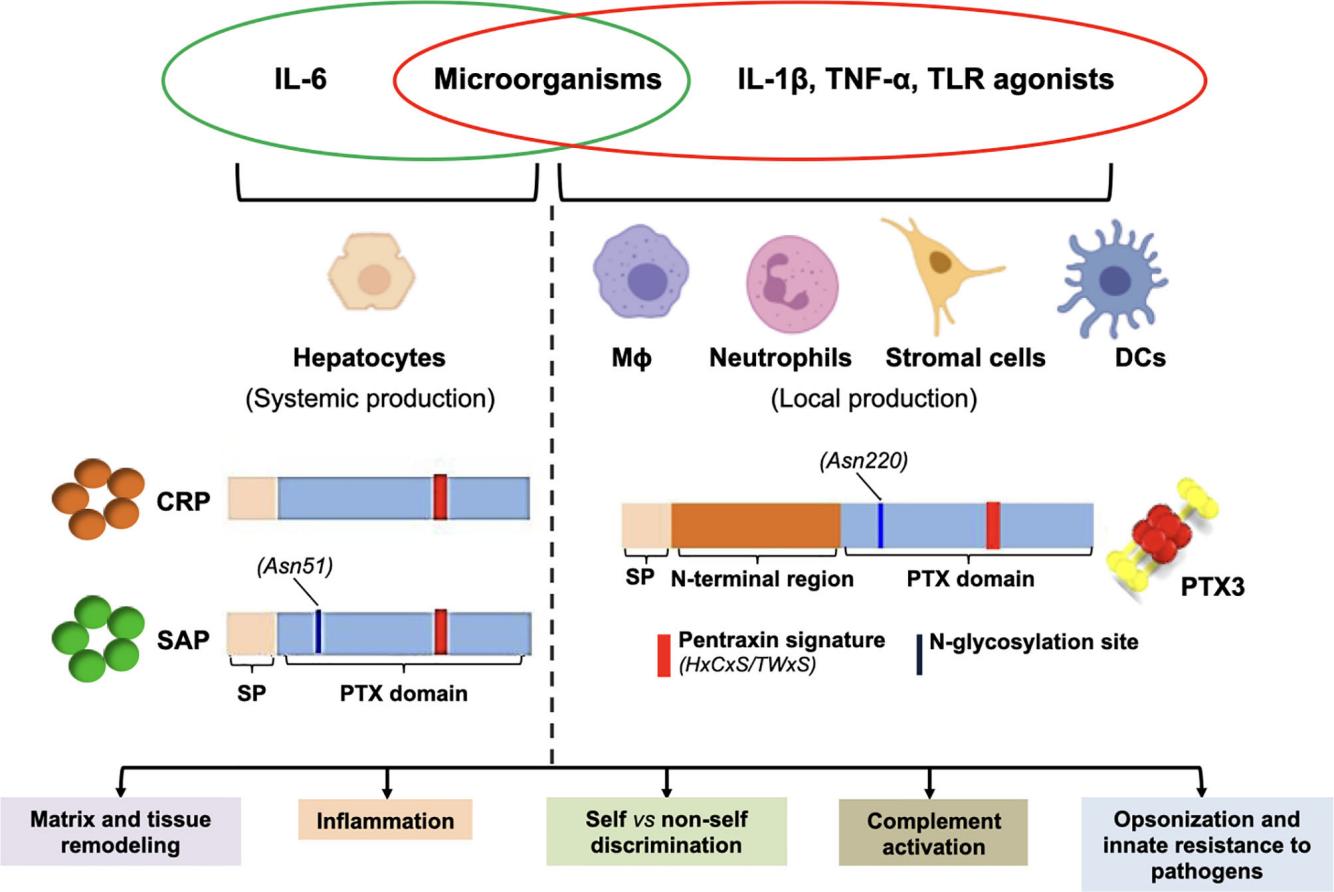
Pentraxins at a glance. Major aspects of the biology of pentraxins are presented here that are further discussed in the main text. The short pentraxins CRP and SAP are mainly synthesized by the hepatocytes in response to IL-6 (systemic production), whereas PTX3 (prototypical long pentraxin) is locally made by a number of myeloid and stromal cells upon stimulation with pro-inflammatory cytokines and/or microbial moieties. All pentraxins share a family distinctive signature within the pentraxin (PTX) domain, and the long ones additionally contain an N-terminal region that is structurally unrelated to other proteins. In spite of diverse protein structure and gene regulation, these molecules have similar biological properties (summarized in the boxes), which highlights complexity and complementarity of this family of PRMs.

As opposed to CRP and SAP, PTX3 is rapidly synthesized and secreted at sites of infection/inflammation by a variety of immune and non-immune cells in response to TLR engagement, microbial moieties, and inflammatory cytokines (28–30). Mature neutrophils do not transcribe the *PTX3* gene, however they store the pre-made protein in specific granules, and promptly release it upon degranulation (31). Similar to CRP and SAP, PTX3 binds C1q, and controls activation of the CP (17, 32). In addition, PTX3 interacts with components of the lectin pathway (LP), including mannose-binding lectin (MBL) (33), ficolin-1 (34) and -2 (35), and promotes LP deposition on AF and *Candida albicans*. Also, in an analogy with CRP and SAP, PTX3 controls excessive complement activation through specific interactions with fH (36) and C4BP (37) (see **Figure 1** for an overview of pentraxins).

## PTX3 in AF Infections

The long pentraxin PTX3 is a PRM with established roles in the innate immune response to selected pathogens, and prognostic/diagnostic potential as biochemical and genetic biomarker in many systemic infections (38), including invasive pulmonary aspergillosis (IPA) (39), and, more recently, COVID-19 (40). Initial evidence of the involvement of PTX3 in the host resistance to AF dates back to 2002, when it was reported that genetic deficiency of *Ptx3* enhances susceptibility to IPA in immunocompetent mice, due to defective recognition of fungal conidia by neutrophils, AMs, and DCs, and biased Th2 responses (41). This phenotype was reverted by administration of the recombinant protein, and a close functional cooperation was established between PTX3, neutrophils, and the complement system (31, 42). These findings have been recapitulated and extended in experimental models of iatrogenic immune suppression (43) and primary immune deficiencies (44), clinical conditions that predispose to IPA. Furthermore, *PTX3* polymorphisms have been associated to reduced systemic levels of the protein and increased risk of IPA in recipients of hematopoietic stem-cell (HSC) (39, 45) and solid organ transplants (46), chronic obstructive pulmonary disease patients (47) and individuals with hematological malignancies (48). Interestingly, this association is lost in conditions of severe neutropenia (49), which further supports the functional link with neutrophils originally foreseen in the mouse (41).

**Figure 2:**
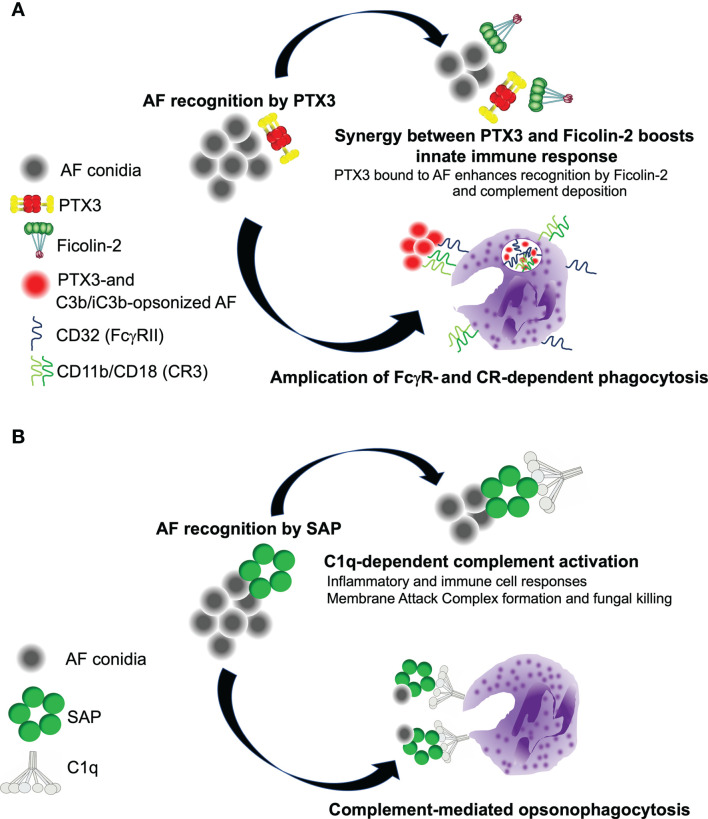
Complement-dependent roles of PTX3 and SAP in the host resistance to *A. fumigatus*. **(A)** PTX3 and Ficolin-2 recruit each other onto AF conidia, and activate the LP. As C3b and iC3b deposit (*via* the AP amplification loop), PTX3 promotes phagocytosis of AF *via* FcγRII (CD32)-dependent redistribution of CD11b (that forms with CD18 the complement receptor 3, CR3, major receptor of iC3b) to the phagocytic cup. **(B)** SAP recruits C1q to AF conidia, and promotes CP activation. This leads to enhanced disposal of the pathogen through neutrophil-dependent phagocytosis and MAC-mediated killing.

The mechanisms underlying the antifungal properties of PTX3 have been addressed in a study by Moalli et al., where this pentraxin was shown to opsonize AF conidia and promote their phagocytosis and killing by human (*in vitro*) and mouse (*in vivo*) neutrophils *via* AP, complement receptor 3 (CR3), and Fcγ receptors (FcγRs) pathways (42). This and previous investigations (41) ruled out contributions of CP (C1q in particular) to the pro-phagocytic activity of PTX3. Also, ficolin-2 and PTX3 have been reported to recruit each other to the wall of AF conidia, and promote synergic amplification of the LP, however this mechanism is relevant in conditions of C1q and MBL deficiency only (35). Therefore, available evidence indicates that a functionally competent AP is required for the pro-phagocytic and pro-killing activities of PTX3 in AF infections [see **Figure 2A** and (10) for a more comprehensive review of the interplay between PTX3 and complement in these diseases].

## SAP and Microbial Pathogens

SAP is recognized as a component of the innate immune response to microbial pathogens, including Gram-positive (50, 51) and Gram-negative bacteria (52) and viruses (53), and traditionally described as an opsonin that acts through FcγRs and complement mechanisms (54–58). However, the actual role of SAP in clinical infections is unclear (52), likely due to divergent gene regulation in mice and humans (16, 21, 59), and conflicting evidence from *in vitro* and *in vivo* settings (52). For example, SAP interacts with spikes on the viral envelope of the influenza A virus, inhibits hemagglutination, and neutralizes virus infectivity *in vitro* (53, 60), however it has no clear role in human influenza (61). Also, in spite of inhibitory effects on the intra-erythrocytic growth of malaria parasites (62) and uptake of *Mycobacterium tuberculosis* by murine AMs *in vitro* (63), no data are available to support a role of SAP in malaria and tuberculosis *in vivo*. Furthermore, a clear correlation between microbial recognition, opsonic activity and microbicidal function of this pentraxin is missing. In this regard, SAP is known to interact with *Streptococcus pneumoniae* and promote its phagocytosis *in vitro* and *in vivo* (51), however it enhances the macrophage-dependent killing of *Listeria monocytogenes*, a pathogen it does not bind (64). Also, this short pentraxin recognizes *Mycobacterium tuberculosis*, and inhibits recognition and killing of this pathogen by macrophages (63). On the same line, its interaction with *Streptococcus pyogenes*, *Neisseria meningitides*, and some strains of *Escherichia coli* results into decreased phagocytosis and killing by macrophages and inhibition of complement, and SAP-deficient mice have increased survival in experimental infections with *Streptococcus pyogenes* or *Escherichia coli* (52). Based on these and other evidence, the opsonic nature of SAP has been questioned (52), and pharmacological depletion rather than administration of SAP has been proposed to treat invasive infections (65). Moreover, the regulatory mechanisms through which SAP participates in the immune response to unligated microbial pathogens are yet to be defined.

## Emerging Roles of SAP in Antifungal Immunity

A functional interaction of SAP with filamentous forms of pathogenic fungi has been proposed (66), based on histology of autoptic specimens from patients with invasive gastrointestinal candidiasis, aspergillosis, mucormycosis, and coccidioidomycosis (67, 68). Also, in a mouse model of chronic AF-induced allergic asthma, administration of SAP inhibited alternative macrophage activation, airway remodeling and inflammation (69). This occurred *via* engagement of FcγRs (70), a mechanism through which SAP controls fibrocyte differentiation in addition to alternative macrophage polarization (71, 72).

SAP is a well-known player in amyloidosis, where it binds and stabilizes amyloid fibrils (73). These form on the surface of invading yeasts and fungi too (68), which suggests that SAP might contribute to the pathogenicity of these microbes by stabilizing amyloid deposits that interfere with immune recognition (67). Indeed, the interaction of SAP with amyloid fibrils on *Candida albicans* inhibits phagocytosis and cytokine production in macrophages (74). However, genetic deficiency of SAP had no effect in a mouse model of candidiasis (11).

We have recently reported that SAP is an essential element of the host resistance to AF and other clinically relevant fungi of the *Trichocomaceae* family, including *A. flavus* and *A. terreus* (11). In a murine model of lung aspergillosis, SAP interacts with AF conidia, and triggers complement-mediated inflammatory responses that are essential for pathogen removal. Indeed, SAP-deficient mice are more susceptible to the experimental infection, due to reduced recruitment and phagocytic activity of neutrophils, and resistance to AF is rescued in these animals by administration of the recombinant murine protein. Also, the recombinant human protein, currently under evaluation for therapy of idiopathic pulmonary fibrosis (IPF) (75–77), has therapeutic efficacy against AF in transiently myelosuppressed mice, an experimental setting that closely mimics iatrogenic IA in humans. More importantly, polymorphisms in the *APCS* gene (coding for the SAP protein) are associated to the risk of IPA (11).

We have shown that SAP binding to AF conidia results into deposition of C3 and production of the anaphylatoxin C5a, which is required for effective recruitment and phagocytic activity of neutrophils in the infected lung (78). Consistent with this, the plasma of *Apcs^-/-^* mice had decreased C3 activation and C5a levels when challenged with AF conidia *in vitro*, and pre-opsonization with the murine protein rescued complement activation and AF phagocytosis by neutrophils from SAP-deficient and -competent mice. Also, in the presence of active complement, bone marrow-derived macrophages from *Apcs^-/-^* animals had reduced production of cytokines when exposed to AF conidia, further strengthening the point that SAP exerts a complement-dependent pro-inflammatory role in IA (11). Based on opsono-phagocytosis experiments with human and mouse sera depleted of selected complement components, we demonstrated that, when opsonized to AF, SAP promotes activation of the CP, a major initiator of complement in AF infections (79) (**Figure 2B**). As opposed to this, the pro-phagocytic activity of PTX3 does not require C1q (and the CP) (41, 42), likely due to the conidia-bound protein being unable to bind C1q and/or induce the structural rearrangements that are needed for this protein to activate the CP (80). Moreover, SAP-mediated induction of complement culminates in the formation on AF conidia of the membrane attack complex (MAC; C5b-C9), and fungal killing, pointing to a complement-dependent microbicidal effect of this pentraxin in the serum (11).

Adaptive immunity plays an important role in fungal infections, whereby anti-AF IgG seroprevalence has been described in geographic areas with prevalence of chronic pulmonary aspergillosis, and anti-AF IgGs have been detected in healthy subjects too (81). Also, neutrophils, major cellular players in IPA, express FcγRs (78), and SAP has been proposed as a ligand of FcγRs (70). Using antibodies to block FcγRs or IgG-depleted plasma, we have indeed documented decreased phagocytosis of AF by neutrophils, suggesting that the IgG/FcγR axis is involved in fungal removal by these cells (11). However, SAP retained its pro-phagocytic activity on neutrophils even in conditions of FcγRs blockade or IgG depletion, indicating that the antibody-mediated engagement of FcγRs is dispensable for the SAP-dependent opsono-phagocytosis of AF. Interestingly, pre-opsonization of conidia with SAP potentiated C3 deposition even in IgG-depleted plasma, a condition that mimics antibody deficiencies in humans. Also, SAP amplified phagocytosis of AF by monocytes and macrophages, in addition to neutrophils, but failed to do so with DCs, possibly due to these cells expressing low levels of complement receptors (82).

We have reported that single nucleotide polymorphisms in the *APCS* gene of HSC donors (rs2808661 and rs3753869 SNPs) are associated with the incidence of IPA in recipients of allogeneic HSC transplants (11). Homozygous for the pathological alleles are relatively rare in the general population, however, these genotypes have a high degree of penetrance with cumulative incidence of infection of ~50%. This suggests that SAP is of pathogenetic relevance in human IPA, and indicates that genetic variation in the *APCS* gene might be clinically valuable, for example, to screen donors in HSC transplantation and identify individuals at high risk of IPA. The genetic association between SAP and IPA is quite surprising, given that the expression of this pentraxin is traditionally confined to the liver (72). However, local expression of SAP has been documented in atherosclerotic (83) and fibrotic lesions (84), and, more importantly, *in silico* analyses have detected *APCS* mRNA in human and murine immune cells (including neutrophils, monocytes and macrophages) in inflammatory conditions. Also, *APCS* is expressed in peripheral monocytes from COVID-19 patients (11). Interestingly, the concentration of SAP increases in the BALF but not in the blood of IPA patients, and lower levels of the protein have been found in the serum of recipients of HSC from donors with the IPA-associated *APCS* genotypes (11), suggesting that SAP is a local rather than systemic player in IPA pathogenesis.

CRP has been reported to increase in the serum of IA patients (85, 86), recognize fractions of the AF hyphal wall (87), and promote AF phagocytosis by human neutrophils *in vitro* (88). However, whether this short pentraxin is involved in the pathogenesis of IA *in vivo* is unknown.

## Discussion

Experimental and clinical evidence indicates that the long pentraxin PTX3 and the short pentraxin SAP are key players in the host resistance to fungal infections, particularly those mediated by *A. fumigatus*. These proteins both exert complement-dependent pro-phagocytic and pro-killing activities, and closely crosstalk to major cellular components of the innate immune system, especially neutrophils. Interestingly, they cooperate with distinct pathways of complement, and exhibit diverse FcγRs requirements (11, 42), which points to integrated and, possibly, complementary roles of the two pentraxins in antifungal immunity. In this regard, PTX3 has been shown to add on or synergizes with clinically established antifungal drugs in several animal models of IPA (43, 89–91). Whether this is the case for SAP too remains to be assessed, however, based on our current mechanistic understanding, it is envisaged that the combination of the two proteins might have additive or synergic effects, and possibly pave the way to new therapeutic options against drug-resistant AF strains (92).

In our model of AF infection in immunosuppressed mice the recombinant human SAP had therapeutic efficacy at comparable doses to those used in lung fibrosis (84) and influenza (93), which fosters translation to prophylaxis and therapy of IPA in immune-compromised patients (94). Also, given the fact that SAP mediates assembly of MAC on AF and fungal killing in the serum, this pentraxin might find therapeutic applications in conditions of neutropenia too. The interaction of SAP with FcγRs is known to inhibit the alternative activation of macrophages (69), which restrains tissue fibrosis *in vivo* (71, 84). Based on this rationale, SAP has been shown to have anti-fibrotic activity in mouse models of chronic diseases of the kidney (95) and lung (84), and in the prophylactic treatment of influenza (93). More importantly, a recombinant form of human SAP (PRM-151) has been proposed as a novel anti-fibrotic immunomodulator in IPF patients, based on phase 2 randomized and placebo-controlled trials, with no serious adverse reactions (75–77), which encourages clinical trials to evaluate the efficacy of this short pentraxin, in addition to PTX3, in the treatment of IA. In an era of COVID-19 pandemic, these translational efforts are timely, given that a strong and independent association has been established between IPA and the COVID-19 disease (96, 97).

## Author Contributions

RP wrote the “PTX3 in AF infections” chapter. VP wrote the “Introduction” chapter. ME wrote the “Pentraxins and their interaction with the complement system” chapter. AD wrote the “SAP and microbial pathogens” and “Emerging roles of SAP in antifungal immunity” chapters. FD, RP, and VP generated the figures. AI conceptualized and outlined the manuscript, wrote the Abstract and the “Discussion” chapter, and revised the manuscript. BB, CG, AM, and AD contributed to critical revision of the manuscript. All authors contributed to the article and approved the submitted version.

## Funding

The authors gratefully acknowledge Fondazione Beppe e Nuccy Angiolini for funding a post-doctoral fellowship (recipient RP) and a technician contract (recipient VP). Most of the work done on PTX3 and SAP in the last years has been funded by the European Commission (ERC project PHII-669415 to AM) and the Italian Ministry of Health (GR-2011-02349539 to AI). We are also grateful to Associazione Italiana Ricerca sul Cancro (AIRC, IG-21714 to CG, and IG-23465 to AM) for the financial support.

## Conflict of Interest

AM and AD are inventors of a patent on SAP (WO2020127471). AI is inventor of a patent on PTX3 (WO2006037744A1). AM, CG, and BB obtain royalties on reagents related to PTX3.

The remaining authors declare that the research was conducted in the absence of any commercial or financial relationships that could be construed as a potential conflict of interest.

## Publisher’s Note

All claims expressed in this article are solely those of the authors and do not necessarily represent those of their affiliated organizations, or those of the publisher, the editors and the reviewers. Any product that may be evaluated in this article, or claim that may be made by its manufacturer, is not guaranteed or endorsed by the publisher.
